# Influenza in patients with cancer after 2009 pandemic AH1N1: An 8‐year follow‐up study in Mexico

**DOI:** 10.1111/irv.12704

**Published:** 2019-11-20

**Authors:** Diego Ángeles‐Sistac, Alexandra Martin‐Onraet, Patricia Cornejo‐Juárez, Patricia Volkow, Carolina Pérez‐Jimenez, Diana Vilar‐Compte

**Affiliations:** ^1^ Department of Infectious Diseases Instituto Nacional de Cancerología Mexico City Mexico

**Keywords:** AH1N1, cancer, human, influenza, sentinel surveillance

## Abstract

**Background:**

Immunosupressed patients are at high risk of influenza‐related complications. Influenza AH1N1 has been hypothesized to induce worse outcomes in patients with malignancies, but after the A(H1N1)pdm09 few publications have analyzed the presentation and complications related to influenza afterward.

**Objectives:**

We aimed to describe the characteristics, risk factors, and outcomes of influenza in an oncologic center after the 2009 pandemic and to compare our case distribution to the National community acquired influenza databases in Mexico and the United States.

**Methods:**

We reviewed the cases of confirmed influenza in patients with cancer from an oncological center in Mexico from April 2009 to April 2017. Data on severity and influenza type, malignancy, comorbidities, and outcomes were recorded. We correlated data between the Centers for Disease Control and Prevention (CDC) in the United States and SISVEFLU (Influenza Surveillance Program) in Mexico.

**Results:**

One hundred eighty‐eight patients were included; 75 (39.9%) had a solid neoplasm and 113 (60.1%) had hematologic malignancies. AH1N1 was the most frequent influenza type (54.2%). Patients with hematologic malignancies had more pneumonia (55% vs 25%, *P* < .001), needed more hospitalizations (75% vs 39% *P* < .001), had higher all‐cause mortality at 30 days (20% vs 9% *P* = .048) and influenza‐associated mortality (17% vs 7% *P* = .041). Thirty (16%) patients died within 30 days, and 24 (12.7%) were related to influenza. Influenza type was not associated with worse outcomes. Yearly occurrence of influenza reported by the CDC and SISVEFLU showed a significant correlation (*ρ* = 0.823, *P* = .006).

**Conclusions:**

AH1N1 was the dominant serotype. Patients with hematologic malignancies had more severe influenza and presented worse outcomes. Annual SISVEFLU and CDC surveillance information showed a similar distribution of cases along time but influenza serotypes did not match for all seasons.

## 1 BACKGROUND

Influenza viruses can spread rapidly throughout the world during epidemics and yearly seasonal outbreaks in regions with temperate weather. During these outbreaks, 5%‐10% of all adults may acquire the disease, causing serious illness in 3‐5 million persons each year, and between 250 000 and 500 000 deaths all over the world.^1^ In immunocompromised patients, influenza closely parallels the occurrence of the infection in the community,^2^ with higher rates of pneumonia and infection‐related mortality.^3^, ^4^, ^5^, ^6^, ^7^, ^8^, ^9^

In 2009, a new pandemic influenza virus A(H1N1)pdm09 was identified in Mexico, which rapidly spread all over the world.^1^, ^4^, ^10^ Among general population, infection with the AH1N1 virus is usually associated with mild respiratory disease with little hospitalization or death^7^, ^8^; however, it has been reported that influenza AH1N1 infection can cause a more severe disease than the seasonal influenza virus.^2^ In Mexico, the AH1N1 virus has been accompanied by higher mortality rates and a cyclic biannual behavior—alternating with the AH3N2 virus.^1^ Globally, during the 2009 A(H1N1)pdm09 pandemic, this virus caused 84.1% of all influenza infections, approximately 18 000 deaths and an overall case‐fatality rate lower than 0.5%.^3^, ^7^, ^10^, ^11^

Patients with cancer and those with immunosuppression are at high risk of influenza‐related complications.^1^, ^6^, ^12^ These patients were particularly prone to develop serious complications and adverse outcomes at the beginning of the A(H1N1)pdm09 pandemic. The AH1N1 strain has been hypothesized to induce worse outcomes in patients with malignancies. Early studies in patients with hematologic malignancies (HM) and hematopoietic cell transplant demonstrated higher rates of hospital admissions, pneumonia, intensive care unit admissions, and mortality^2^, ^3^, ^7^, ^8^, ^10^; nonetheless, there is insufficient evidence on whether this strain induces worse outcomes.^8^, ^13^ Few studies have reported no difference in outcomes when comparing AH1N1 and other types of influenza in patients with cancer.^2^, ^4^, ^10^

During the peak of influenza season, the rate of pneumonia in patients with cancer varies between 21% and 80%, and fatality rates range from 11% to 38%.^4^, ^8^, ^9^, ^14^, ^15^, ^16^ Patients with HM and bone marrow transplant remain at high risk of severe influenza infections.^8^, ^13^, ^15^, ^17^, ^18^ The underlying disease, chemotherapy related toxicity, immunosuppression caused by medications and poor vaccination rates are all contributing factors for infection and severe forms of the disease in this subset of patients.

After the A(H1N1)pdm09 pandemic, limited studies on cancer patients were published^5^, ^8^, ^15^, ^16^, ^19^ and very few publications have reported the presentation and complications related to influenza afterward. The aim of this study was to describe the epidemiology, characteristics, and outcomes of influenza of eight consecutive years in a cohort of patients with cancer and to compare the yearly trends for the same time lapse with community acquired influenza data published by the United States’ Centers for Disease Control and Prevention (CDC) and Mexico's Influenza Surveillance (SISVEFLU).

## 2 METHODS

### 2.1 Setting and design

We conducted a retrospective analysis of patients with cancer and confirmed influenza at the Instituto Nacional de Cancerologia (INCan) during the study period. INCan is a 136‐bed referral, teaching hospital for adolescents and adult patients with cancer, in Mexico City. Since the A(H1N1)pdm09 pandemic a prospective surveillance program for patients with influenza‐like illness was established, all hospitalized patients and those with a high suspicion of influenza are evaluated by an Infectious Diseases specialist.

### 2.2 Patients

All patients with solid or HM and laboratory‐confirmed influenza who received care at INCan from April 1, 2009, to March 31, 2017, were included.

We searched the influenza database for all patients with cancer and confirmed diagnosis of influenza, along with the electronical medical record of each patient. We collected the following data: demographics (age and gender), comorbidities, underlying malignancy, oncological treatment, clinical presentation, radiologic characteristics, laboratory data, co‐infections, antiviral treatment, and outcomes.

### 2.3 Definitions

A case of influenza was defined as a patient with either a solid malignancy or an HM (leukemia, lymphoma, multiple myeloma, and others) having acute respiratory illness and a positive real‐time polymerase chain reaction (PCR) assay. The infection was classified as community acquired if the onset of symptoms occurred before or within the first 2 days after hospital admission, or nosocomial if the symptoms developed any time after that during hospitalization. Upper respiratory infection (URI) was characterized by onset of rhinorrhea, nasal/sinus congestion, pharyngitis, or cough with or without expectoration, and its diagnosis was confirmed by PCR. Lower respiratory infection (LRI) or pneumonia was characterized by new or changing pulmonary infiltrates suggestive of viral etiology on chest imaging and confirmed via examination of respiratory specimen, including nasal washes, endotracheal tube aspirates, sputum specimens, and bronchoalveolar lavage fluid specimens. A co‐infection was considered when another organism was isolated from a patient at the time of influenza diagnosis. A concurrent infection was defined when another organism was isolated from a patient within 3 days after the diagnosis of influenza. Non‐infectious complications were also considered. Acute kidney injury was defined as specified by the AKIN classification (*ie*, sudden decrease [in 48 hours] of renal function, expressed by an increase in absolute serum creatinine of at least 0.3 mg/dL). Hypoxia was defined as ambient air blood oxygen saturation <90% by pulse oximetry at diagnosis. Severe neutropenia was considered as an absolute neutrophil count <500 cells/mL and severe lymphocytopenia as an absolute lymphocyte count <200 cells/mL. Elevated creatinine was defined as creatinine serum levels over 1.2 mg/dL and hypoalbuminemia as albumin serum levels <3.5 g/dL.

### 2.4 Statistical analysis

Statistical analysis was performed with Stata version 12.0 (StataCorp LP). Categorical variables were compared using the *chi‐square test* or Fisher's exact test. Continuous variables were compared using the Mann‐Whitney *U* test or the Student's *t* test. Logistical regressions were conducted for all outcome categorical variables including known risk factors to obtain Odds Ratios on both, unadjusted and adjusted models. Multiple Kaplan‐Meier survival analysis was performed, and Log‐rank test results were included when group comparison was needed. Finally, we used Pearson's correlations to measure associations between the CDC's, SISVELU's, and our own yearly influenza reports. All reported *P* values were two‐sided and set with significance levels at <.05.

## 3 RESULTS

### 3.1 Baseline characteristics

We identified 188 patients with laboratory‐confirmed influenza during the study period; one patient was reported to have influenza infection in two different years. Median age was 47 years (range 15‐79 years), and 99 (52.6%) patients were female. One hundred thirteen patients (60.1%) had a hematologic malignancy, 51 (45.1%) were lymphomas, 32 (28.3%) were leukemia, and 23 (20.3%) were multiple myeloma. Seventy‐five patients (39.9%) had a solid malignancy, being breast cancer the most frequent with 42 (56%) cases, followed by cervical cancer (n = 6, 8%) and lung cancer (n = 3, 4%). One hundred and fifteen (61.1%) patients received chemotherapy within 30 days of influenza diagnosis. Obesity was the most common comorbidity, affecting 49 (26%) patients. Vaccination rates were low, with only 18 (9.5%) patients being vaccinated against influenza. LRI was reported in 81 (43%). One hundred and eighty (95.7%) patients were treated with oseltamivir (1 patient received zanamivir). Patients characteristics are depicted in Table 1.

**Table 1:** Clinical characteristics and outcomes of patients with influenza

Variable	Total n = 188 N (%)	Hematologic malignancies n = 113 N (%)	Solid neoplasia n = 75 N (%)	*P* value
Median age, years (range)	47 (15‐79)	45 (15‐79)	49 (20‐78)	.167
Sex				
Male	89 (47)	75 (66)	14 (19)	<.001
Female	99 (53)	38 (34)	61 (81)	
Chemotherapy within the last month	115 (62)	73 (65)	42 (56)	.206
Comorbidities				
HIV	15 (8)	12 (11)	3 (4)	.094
Diabetes	20 (11)	12 (11)	8 (11)	.992
Active smoker	11 (6)	7 (6)	4 (5)	.805
Chronic kidney disease	5 (3)	4 (4)	1 (1)	.357
Cardiovascular	34 (18)	19 (17)	15 (20)	.578
Obesity	49 (26)	27 (24)	22 (29)	.317
Vaccinated against Influenza	18 (10)	9 (8)	9 (12)	.642
Infection site at diagnosis				
Upper respiratory tract	107 (57)	51 (45)	56 (75)	<.001
Lower respiratory tract	81 (43)	62 (55)	19 (25)	
Oseltamivir	180 (96)	109 (96)	71 (95)	.830
Median length of time from symptoms to oseltamivir, days (range)	3 (0‐28)	3 (0‐28)	2 (0‐6)	.069
Hospital admission	114 (65)	85 (75)	29 (39)	<.001
All‐cause mortality at 30 d	30 (16)	23 (20)	7 (9)	.048
Influenza related mortality	24 (13)	19 (17)	5 (7)	.041

**Figure 1:**
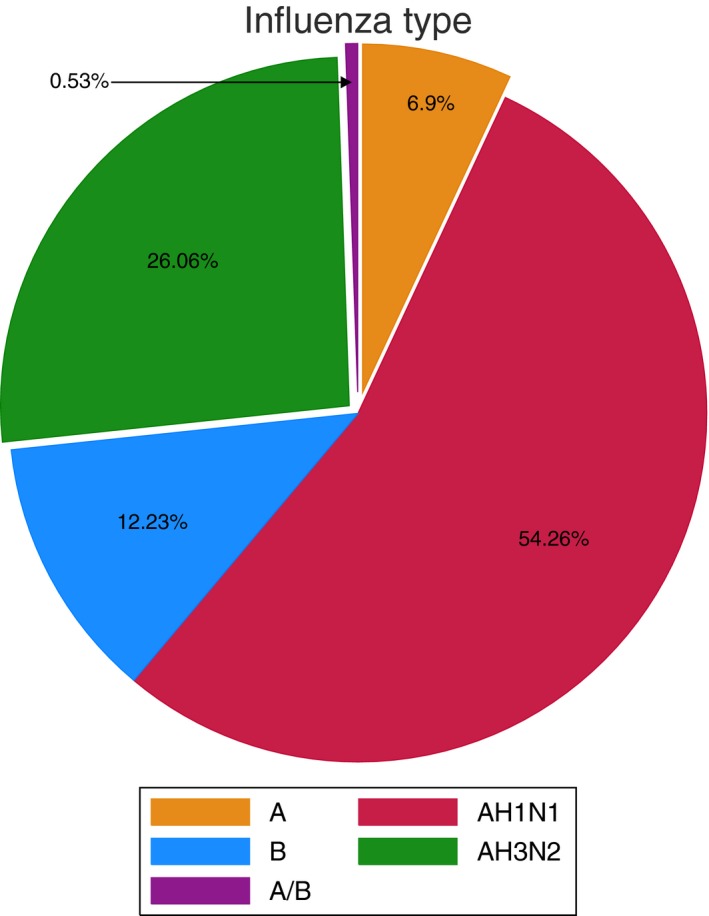
Influenza serotypes in the study population

Influenza AH1N1 was the most common influenza type across all years (54.2%), followed by AH3N2 (26%) and influenza B (12.2%) (Figure 1). The most frequent presenting symptoms were cough (94.6%), fever (87.2%), and general malaise (80.8%). HM patients presented with higher rates of fever (94% vs 70% solid malignancies, *P* = .04), but a similar hierarchy of other symptoms was observed between patients with HM and solid malignancies.

Median length of time from influenza‐like symptoms to antiviral treatment was 4 days (0‐28 days), despite the large range, only seven patients received treatment more than 5 days after the onset of symptoms. One hundred and fourteen (60.6%) patients were hospitalized. Thirty (16%) patients died within the 30 days of influenza diagnosis; 24 (12.7%) were associated with influenza. Patients with HM had more LRI (55% vs 25%, *P* < .001), were hospitalized more frequently (75% vs 39% *P* < .001), and had higher all‐cause 30‐day mortality (20% vs 9% *P* = .048) and higher influenza‐related mortality (17% vs 7% *P* = .041).

From the 114 patients that required hospitalization (Table 2), hypoxia was reported in 47 (41.2%), severe lymphopenia in 29 (25.4%), severe neutropenia in 22 (19.2%), elevated creatinine in 17 (14.9%) and hypoalbuminemia in 69 (60.5%). Nosocomial pneumonia due to influenza was documented in 18 (15.7%) patients; 15 (83.3%) had a HM and 3 (16.6%) a solid neoplasm. Fourteen of all nosocomial cases (77.7%) were caused by influenza AH1N1.

**Table 2:** Variables related to patients with influenza‐related pneumonia

Variable	Total (n = 114)	Hematologic neoplasia (n = 85)	Solid malignancies (n = 29)	*P* value
Hypoxia (Sat O_2_ < 90%)	47 (41)	35 (41)	12 (41)	.487
Severe Lymphopenia, ≤200 cells/mm^3^	29 (25)	26 (31)	3 (10)	.023
Severe Neutropenia ≤500 cells/mm^3^	22 (19)	20 (24)	2 (7)	.046
Elevated creatinine >1.2 mg/dL	17 (15)	14 (16)	3 (10)	.499
Hypoalbuminemia, <3.5 g/dL	69 (60)	55 (65)	14 (48)	.303
Co‐infection at admission	11 (10)	10 (12)	1 (3)	.190
Nosocomial influenza	18 (16)	15 (18)	3(10)	.352
Non‐infectious complication				
Acute Kidney Injury	20 (18)	16 (19)	4 (14)	.380
Other	4 (3)	4 (5)	0 (0)	
ICU admission				
ICU admission within the first 24 h	8 (7)	6 (7)	2 (7)	.976
ICU admission after 24 h	18 (16)	15 (18)	3 (10)	.352
Mechanical ventilation	33 (29)	28 (33)	5 (17)	.107
Median length of hospital stay, days (range)	12 (1‐66)	14 (1‐66)	9 (1‐32)	.057
Median length of ICU stay, days (range)	14 (1‐65)	15 (1‐65)	13 (7‐21)	.817
Median length of MV, days (range)	13 (1‐65)	13 (1‐65)	13 (6‐19)	.974
Median length of time from symptoms to oseltamivir, days (range)	4 (0‐28)	5 (0‐28)	3 (1‐6)	.219

Co‐infection at diagnosis occurred in 23 (12.2%) cases; reported co‐infections were both single and mixed infections, including virus, bacteria, and fungus: *Chlamydophila pneumoniae* (n = 7), *Haemophilus influenzae* (n = 5), Rhinovirus (n = 5), *Streptococcus pneumoniae* (n = 4)*, Bordetella pertussis* (n = 2), Respiratory Syncytial Virus (n = 2), Adenovirus (n = 2), Metapneumovirus (n = 2), Methicillin‐Susceptible *Staphylococcus aureus* (MSSA) (n = 1), and *Aspergillus* spp. (n = 1).

During hospitalization, 19 patients (16.6%) developed other infectious complications (Table S1); 17 patients had pneumonia caused by *Aspergillus* spp. (n = 4), *Pseudomonas aeruginosa* (n = 3, one of which exhibited Multidrug Resistance [MDR]), and *Klebsiella pneumoniae* (n = 3, one with Extended‐Spectrum beta‐lactamases). Seven patients also developed bacteremia: Methicillin‐Resistant *S aureus* (MRSA) (n = 2, one of which also had positive blood cultures for *S* *saprophyticus*); MDR *A baumannii* (n = 1); MSSA (n = 1); methicillin‐resistant *S haemolyticus* (n = 1); *S epidermidis* (n = 1); and *Enterococcus faecium* (n = 1). Other infections included: bacterial conjunctivitis (n = 1), herpes zoster (n = 1), and oral candidiasis (n = 1).

Twenty‐four (21%) patients, all with influenza‐related pneumonia developed a non‐infectious complication, being AKI the most common (n = 20, 83.3%); one patient with AKI also developed concurrent rhabdomyolysis; and another patient developed a right basal ganglia stroke during hospitalization.

Twenty‐six (22.8%) patients were admitted to the intensive care unit (ICU) and 8 (30.7%) within 24 hours of admission. From the group of patients with influenza‐related pneumonia, thirty‐three (28.9%) required mechanical ventilation (MV), some of them were ventilated outside the ICU. Patients with HM had higher rates of severe lymphopenia (31% vs 10% *P* = .023) and neutropenia (24% vs 7% *P* = .046) (Table 2).

### 3.2 Outcome analysis

Risk factors associated with pneumonia after adjusting for dyspnea, lymphocytopenia, and creatinine >1.2 mg/dL are shown in Table 3. Mechanical ventilation (OR, 48.20 [95% CI, 3.58‐648.15]; *P* = .003) was the only variable associated with mortality by multivariate analysis. Patients that at influenza diagnosis were co‐infected with either virus, bacteria, or fungus did not have worse outcomes.

**Table 3:** Risk factors associated with pneumonia by logistic regression analysis

Variable	OR (95% CI)	*P* value
Hematologic malignancy	3.40 (1.12‐10.33)	.031
Hypoxemia at diagnosis	5.44 (1.69‐17.46)	.004
Hypoalbuminemia	7.60 (2.53‐ 22.86)	<.001

Note

Risk factors associated with pneumonia were adjusted for dyspnea, lymphocytopenia, and creatinine >1.2 mg/dL in the logistic regression analysis.

### 3.3 Survival analysis

**Figure 2:**
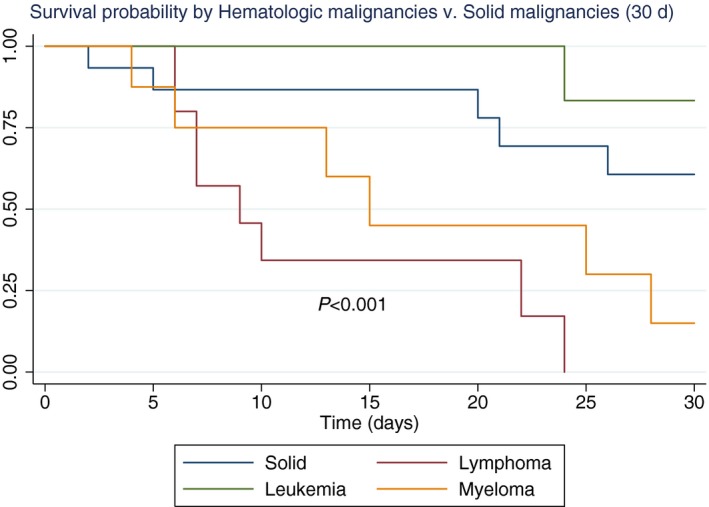
Thirty‐days survival probability in patients with influenza and solid or hematologic malignancies

**Figure 3:**
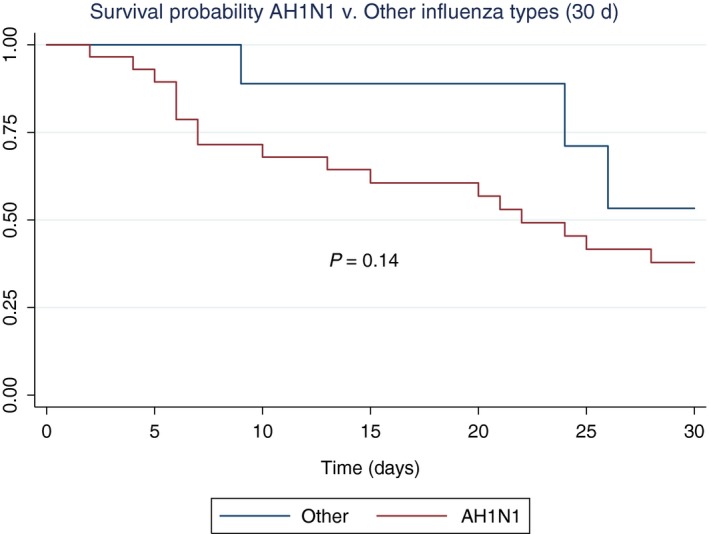
Survival probability at 30 d in patients with influenza AH1N1 compared with other types

We performed Kaplan‐Meier curves on survival at 30 days. Patients with solid malignancies had a greater survival probability than patients with HM, except for patients with leukemia (*P* < .001) (Figure 2). Survival probability of patients with AH1N1 did not differ from those with other types of influenza (*P* = .14) (Figure 3). Treatment with oseltamivir <48 hours after initiation of symptoms did not change the survival probability (*P* = .85) as shown in Figure S1.

### 3.4 Influenza trends

**Figure 4:**
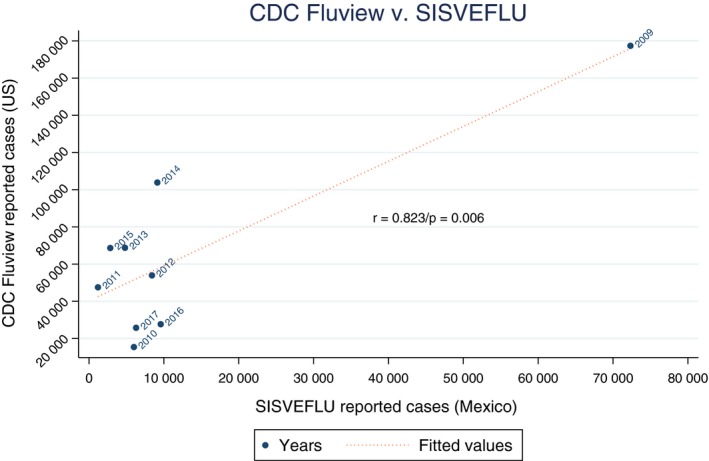
Data on influenza from the USA CDC and SISVEFLU (Mexico) organized on a yearly basis and compared using a Pearson's correlation

Yearly occurrence of influenza cases reported by the CDC followed a similar trend to that reported by the SISVEFLU in Mexico, with a significant correlation between both data sets (*ρ* = 0.823, *P* = .006) (Figure 4). Similarly, cases of influenza occurring in our hospital (omitting A(H1N1)pdm09 cases, when INCan's active influenza case finding and surveillance started) followed the same trend as those reported by SISVEFLU, although it failed to reach statistical significance (*ρ* = 0.683, *P* = .061). Even if frequency of cases reported by SISVEFLU and the CDC seem to hold a positive association, prevailing influenza serotypes are significantly different; Mexico has presented an almost perfect yearly alternating behavior between AH1N1 (2009, 2012, 2014, and 2016) and other influenza serotypes, mainly AH3N2 (2010, 2011, 2013, and 2015). However, the CDC has only reported a higher prevalence of AH1N1 on two non‐consecutive years: 2009 and 2016.

## 4 DISCUSSION

In this study, we showed that oncologic patients, particularly, patients with HM had more severe forms of influenza, as has previously been described.^15^ They also showed lower probability of survival compared with patients with solid neoplasm. No significant differences between years or subtype of influenza were observed, and influenza A H1N1 was not associated with worse outcomes. The season‐to‐season variability of influenza is well described, but only a few studies analyzed various consecutive years and outcomes related to influenza in patients with HM and solid tumors.

Symptoms presented by our patients were similar to those described in non‐immunosuppressed patients,^3^, ^4^, ^5^, ^7^, ^11^, ^13^, ^15^, ^20^ despite it has been suggested that immunocompromised patients might develop severe influenza without classical symptoms. Cough, fever, malaise with bone and muscle pain, rhinorrhea, and chills were frequent complaints. Patients with HM presented with more fever than patients with solid tumors.

In this cohort, patients with HM, lower albumin levels, and hypoxia at diagnosis were at an increased risk for LRI in the multivariable analysis. Pneumonia was developed in 43%, with a higher proportion in patients with HM (55% vs 25%) (*P* < .001). HM patients presented more frequently with severe lymphopenia (HM 31% vs solid malignancies 10%, *P* = .023) and severe neutropenia (HM 24% vs solid malignancies 7%, *P* = .046), consistent with data from other series^3^, ^4^, ^8^, ^10^, ^11^, ^12^, ^13^; however, this association was not significant in the multivariable logistic analysis.

Decreased albumin level has been associated with LRI.^5^, ^10^ In this series, hypoalbuminemia was associated with pneumonia, which in part reflects the chronic and more advanced stage of disease presented in patients with cancer at our Institution. We also found that hypoxia was as an independent risk factor associated with pneumonia, a characteristic that has previously been described in patients with HM.^5^ Hypoxia has also been linked with greater mortality^5^, ^8^; but in our study, it was not a predictor of death by multivariate analysis. It is difficult to explain the lack of association between hypoxia and mortality but it is probably related to the fact that other variables such as the underlying malignancy and its advanced stage in most patients remained the most important predictors of mortality. Hypoxemia is probably a marker of severe disease, but not a predictor of adverse influenza‐related outcomes.

Obesity has been described as a risk factor for influenza, along with severe complications and death. Also, obese patients shed influenza virus for longer periods of time compared with non‐obese individuals.^21^ In our cohort, although obesity was prevalent, it did not increase the risk of pneumonia or death, but was correlated with dyspnea and hypoxemia, which is consistent with ventilatory dysfunction and increased risk of mechanical ventilation and death.

AKI occurred in 20 (17.5%) patients. Although it has been associated with influenza, this complication has not frequently been described in other studies that included patients with cancer.^8^ The increased rate of AKI in this series may be part of a spectrum of influenza complications in complex patients with advanced oncologic disease.

Delayed initiation of antiviral therapy has been correlated with pneumonia and mortality.^2^, ^6^, ^7^, ^10^, ^16^ In this analysis, patients that received oseltamivir after 3 days had a greater influenza‐related mortality only on the univariate model, but the same variable had no effect on the development of pneumonia. Likewise, in the survival analysis, probability at 30 days was unaffected by the delayed initiation of antiviral therapy; 58.5% of our patients received oseltamivir during the first 3 days of symptoms onset.

Overall, influenza mortality in patients with cancer varies between 22% and 40%.^11^, ^16^ In our cohort, 30‐day mortality was 16% (n = 30) and influenza‐related mortality was 12.7% (n = 24). Most of these deaths occurred in the HM group (19 vs 5 solid malignancies, *P* = .041), reflecting the fragility of these severely immunocompromised patients. Different to what was initially reported with influenza AH1N1, mortality was not increased in patients with A(H1N1)pdm09.^2^, ^3^, ^4^, ^7^, ^8^, ^10^ In this study, AH1N1 was not associated with the development of pneumonia and was only associated with greater mortality in the univariate model. Also, in patients with HM mortality was higher within the AH1N1 group (AH1N1 patients 17 vs non‐AH1N1 2, *P* = .002) while there were no differences in the solid malignancies group. We did not find any differences in survival probability at 30 days when comparing AH1N1 to other influenza types or viral or bacterial co‐infections. Patients with lymphoma and multiple myeloma showed the highest mortality.

Nosocomial influenza has been associated with worse outcomes in patients with cancer. In this series, nosocomial influenza occurred in 18 (15.7%) patients, mostly in HM (15 cases [83.3%]), and it accounted for 8 of the 24 (33.3%) cases of influenza related mortality (*P* < .001), highlighting the importance of increased awareness and rapid implementation of control measures.

It is also relevant to emphasize the importance of high influenza vaccination rates among health‐care workers, as this measure has demonstrated to diminish the risk of nosocomial influenza in patients with cancer and other frail populations.^22^ In our Institution, health‐care workers influenza vaccination is modest (around 53%), and although not quantified, a few cases of nosocomial influenza were transmitted from unvaccinated health‐care workers.

As an original result from our analysis, we investigated a possible correlation between yearly national cases of influenza (SISVEFLU) and our data, and the cases occurring in the United States, in agreement to the proposed relationship between circulation of influenza in immunocompromised patients and general population from the northern hemisphere.^18^ As shown in Figure 4, there was a correlation between the number of influenza cases occurring in the United States and Mexico; however, the magnitude of the association was mostly determined by the A(H1N1)pdm09 pandemic. Also, the CDC's reports show a yearly and continuous larger proportion of AH3N2 in the United States in comparison with Mexico's influenza subtypes, which alternate biannually between AH1N1 and AH3N2.^1^ The less frequent occurrence of H3N2 and an almost perfect yearly alternating behavior between AH1N1 clearly deserves a further and more detailed analysis between the association of subtypes found in Mexico and the United States.

Our study has several limitations since it is a single center observational study, and although most data were collected prospectively, the analysis was conducted retrospectively. Despite the fair number of patients with influenza and cancer, and information from 8 years, we still included more patients with influenza AH1N1 than H3N2 or influenza B, which probably decreased the power to find differences between the outcomes caused by the different types of influenza. Our study might also have some selection bias, as ambulatory patients with mild influenza were probably treated symptomatically with no further testing for influenza. There is also the possibility that patients with mild symptoms requested medical attention in a different health‐care facility. Despite these limitations, this series shows the spectrum of influenza in patients with cancer across a long period of time in Mexico since 2009 when A(H1N1)pdm09 showed up in our country; we also compared our data with that of our national and the CDCs’ surveillance system, a correlation that shows the annual trends of influenza seen in hospitals from Mexico City, and much likely, those located on the central and northern of Mexico, with temperate climates.

In conclusion, influenza AH1N1 was the dominant serotype of influenza in our patients, as it has been in the country since the A(H1N1)pdm09 pandemic with biannual peaks. SISVEFLU surveillance information was correlated with that of the CDC. Patients with hematologic malignancies had more severe influenza and presented worse outcomes, including a high‐associated mortality. Influenza vaccination was low in this cohort despite being recommended in many international and national guidelines. The biannual presentation of influenza AH1N1 in Mexico and the less frequent occurrence of H3N2 compared with other countries deserves further investigation.

## CONFLICT OF INTEREST

Nothing to disclose.
