# Supplementary material for: Influenza in patients with cancer after 2009 pandemic AH1N1: An 8‐year follow‐up study in Mexico
Source: Influenza Other Respir Viruses. 2019 Nov 20;14(2):196–203. doi: 10.1111/irv.12704 (PMC7040981; doi:10.1111/irv.12704)
Supplement: Supplementary file 1 [file IRV-14-196-s001.docx]

**Supplementary table 1.** Microorganisms isolated from a respiratory or blood source in patients with influenza

| **Microorganism** | **Respiratory isolates** | **Blood isolates** |
| --- | --- | --- |
| *Acinetobacter baumannii* MDR | 1 | 1 |
| *Enterococcus faecium* | 0 | 1 |
| *Candida Spp.* | 1 | 0 |
| MR *Staphylococcus haemolyticus* | 1 | 1 |
| MS *Staphylococcus aureus* | 2 | 1 |
| *Serratia Marscescens* ESBL | 1 | 0 |
| *Stenotrophomonas maltophilia* | 2 | 0 |
| *Staphylococcus saprophyticus* | 0 | 1 |
| *Klebsiella pneumoniae* ESBL | 1 | 0 |
| *Aspergillus spp.* | 4 | 0 |
| *Klebsiella pneumoniae* | 1 | 0 |
| *Pseudomonas aeruginosa* | 2 | 0 |
| *Pseudomonas aeruginosa* MDR | 1 | 0 |
| MR *Staphylococcus aureus* | 0 | 2 |
| *Staphylococcus epidermidis* | 0 | 1 |

MDR= Multidrug resistance, MR= Methicillin-resistant, MS= Methicillin-sensitive,

ESBL = Extended spectrum beta-lactamases.

*Note: some patients had multiple isolates.*

**Supplementary figure 1.** 30-days survival probability of patients treated with oseltamivir within and after 48 hours of diagnosis.


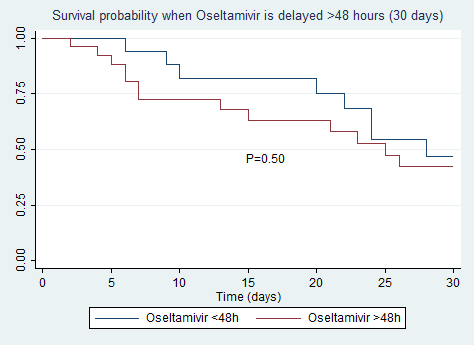


*Color should be used for this figure in print*
